# Chronic Left Gastric Artery and Acute Superior Mesenteric Artery Dissections During a Hypertensive Crisis

**DOI:** 10.7759/cureus.114979

**Published:** 2026-08-22

**Authors:** Victor D Ivancev, Cory O'Gara, Stephen Howard, Tarig Mohamed, Amir Hanafi

**Affiliations:** 1 Internal Medicine, Lake Erie College of Osteopathic Medicine, Erie, USA; 2 Internal Medicine, Rochester Regional Health (RRH) Unity Hospital, Rochester, USA

**Keywords:** arterial aneurysm, arterial dissection, hypertension, left gastric artery, spontaneous visceral artery dissection, superior mesenteric artery

## Abstract

Spontaneous visceral artery dissections are rare and poorly understood presentations. Isolated superior mesenteric artery (SMA) and left gastric artery (LGA) involvement is exceptionally uncommon. Concurrent dissections are exceedingly anomalous and diagnostically challenging. We present a 74-year-old man in hypertensive crisis with severe abdominal pain. Computed tomography angiography revealed a chronic LGA dissection with aneurysm and an SMA dissection. Despite high-risk vascular findings, he was stabilized with aggressive blood pressure control and antithrombotic therapy. This case highlights the importance of early imaging and management in patients who present in hypertensive crisis with abdominal pain out of proportion to examination.

## Introduction

A spontaneous isolated visceral artery dissection (SIVAD) is a primary dissection of the layers of a visceral artery wall that occurs without aortic involvement and without any identifiable precipitating cause [1]. An SIVAD most frequently involves the superior mesenteric artery (SMA), which supplies a major portion of the small intestine and proximal colon; its rate of occurrence has risen in recent decades due to increased use of advanced cross-sectional imaging [2]. Nonetheless, its incidence is estimated at less than 0.06% of all cases presenting with acute abdominal pain [3]. Despite the growing recognition of SMA involvement, the spontaneous dissection of other visceral arteries is far less common. For instance, dissection of the left gastric artery (LGA), which provides the essential arterial supply to the stomach, is an exceptionally rare clinical entity, described in only a small number of published cases.

Hypertension is a recognizable risk factor for visceral artery dissection, as chronic elevation in arterial pressure increases shear stress and predisposes to intimal injury [4]. Other underlying mechanisms that contribute to structural vulnerability include atherosclerosis, connective tissue disease, fibromuscular dysplasia, and segmental arterial mediolysis [5]. The intimal injury forms a transverse tear that permits blood to enter a false lumen, creating an intramural hematoma, which can lead to chronic dissection with partial thrombosis and vessel wall remodeling. A hypertensive crisis, defined as severe acute hypertension with either a systolic blood pressure (SBP) of ≥180 mm Hg or a diastolic blood pressure of ≥120 mm Hg, may further precipitate the process of dissection by increasing shear stress on the vulnerable arterial wall [6]. Furthermore, the degenerative changes exhibited by the localized wall stress may predispose the false lumen to expansion and the formation of a dissecting aneurysm [7].

Clinically, these dissections may remain asymptomatic or present with sudden, diffuse abdominal pain that is notably disproportionate to physical examination findings [8]. Failure to recognize that such a presentation could be indicative of SIVAD may result in catastrophic bowel ischemia or aneurysmal rupture. Although spontaneous isolated SMA dissection has been increasingly recognized, simultaneous involvement of the LGA and SMA, particularly with chronic LGA dissection associated with aneurysmal change and acute SMA dissection, remains exceptionally rare and lacks well-established management strategies. We present a case of concurrent chronic LGA and acute SMA dissections identified during the evaluation of severe abdominal pain in the setting of a hypertensive crisis, highlighting the importance of early diagnostic imaging and targeted medical therapy.

## Case presentation

**Figure 1:**
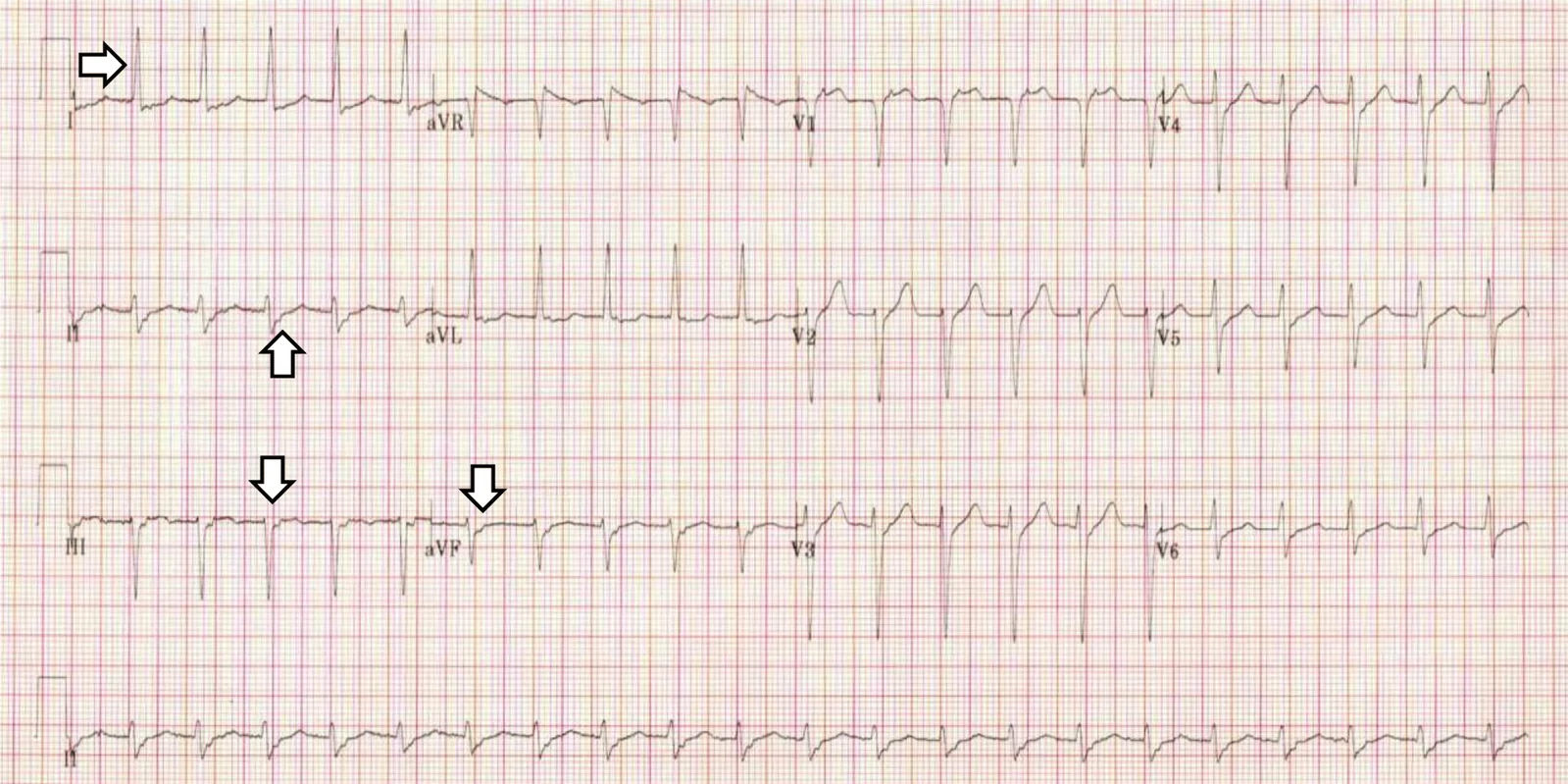
Initial ECG performed in the emergency department revealing a narrow-complex SVT and retrograde P waves aVR: augmented vector right; aVF: augmented vector foot; aVL: augmented vector left; SVT: supraventricular tachycardia; ECG: electrocardiography

A 74-year-old man with a history of primary hypertension, latent syphilis, prior intracranial hemorrhage, and medication nonadherence presented to the emergency department with acute, nonradiating periumbilical abdominal pain with no known aggravating or relieving factors that progressed to diffuse discomfort over the course of 24 hours. The patient described the pain to be sharp, constant, and associated with a sensation of abdominal fullness. He denied any further symptoms such as nausea, vomiting, diarrhea, hematochezia, or recent trauma. He was found to be markedly hypertensive with a blood pressure of 170/130 mmHg and borderline bradycardic. He was afebrile, and oxygen saturation was normal on room air. Laboratory evaluation revealed no abnormalities in the complete blood count, metabolic panel, or lactate, with mildly elevated inflammatory markers (Table 1). Physical examination revealed a soft abdomen with diffuse tenderness to palpation without rebound or guarding, present bowel sounds, and no signs of peritonitis. Cardiopulmonary examination was unremarkable, and peripheral pulses were intact and symmetric. Electrocardiography performed in the emergency department overnight revealed a narrow-complex supraventricular tachycardia (SVT) with retrograde P waves (Figure 1). The SVT spontaneously converted to normal sinus rhythm without intervention the following day and did not alter immediate management.

**Table 1:** Initial laboratory evaluation during emergency department presentation

Test	Reference range	Test result
White blood cell count	4.0-10.0 × 10³/µL	5.7 × 10³/µL
Hemoglobin	13.0-17.5 g/dL	13.6 g/dL
Mean corpuscular volume	81-99 fL	80 fL
Platelet count	150-450 × 10³/µL	162 × 10³/µL
Erythrocyte sedimentation rate	0-20 mm/hour	68 mm/hour
C-reactive protein	0.0-8.0 mg/L	17.2 mg/L
Protime	10.2-12.9 seconds	12.0 seconds
International normalized ratio	0.9-1.1	1.0
Activated partial thromboplastin time	25.1-36.5 seconds	30.2 seconds
Sodium	135-145 mmol/L	135 mmol/L
Potassium	3.5-5.1 mmol/L	4.0 mmol/L
Chloride	98-107 mmol/L	98 mmol/L
Carbon dioxide	20-31 mEq/L	27 mEq/L
Blood urea nitrogen	9-23 mg/dL	18 mg/dL
Creatinine	0.6-1.1 mg/dL	1.0 mg/dL
Glucose	70-99 mg/dL	142 mg/dL
Calcium	8.3-10.6 mg/dL	8.5 mg/dL
Aspartate aminotransferase	7-37 U/L	49 U/L
Alanine aminotransferase	10-49 U/L	29 U/L
Lactic acid	0.4-1.9 mmol/L	0.9 mmol/L

**Figure 2:**
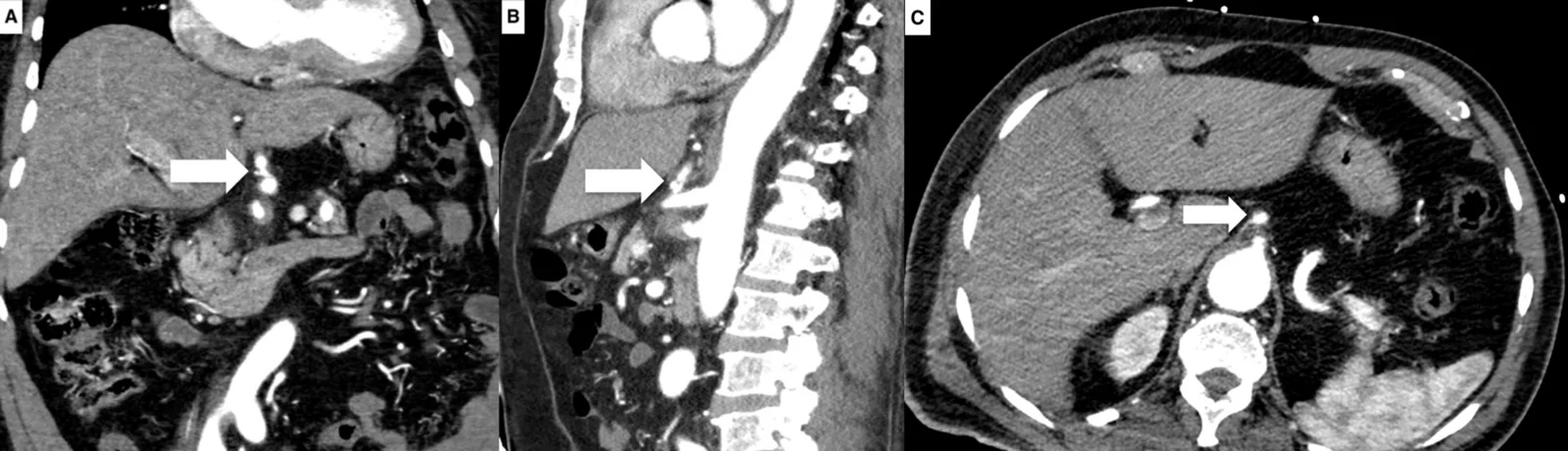
Initial CTA of the abdomen and pelvis obtained on the day of admissions demonstrating a left gastric artery dissection. (A) Coronal, (B) sagittal, and (C) axial views showing the intimal flap and luminal irregularity consistent with dissection CTA: computed tomography angiography

**Figure 3:**
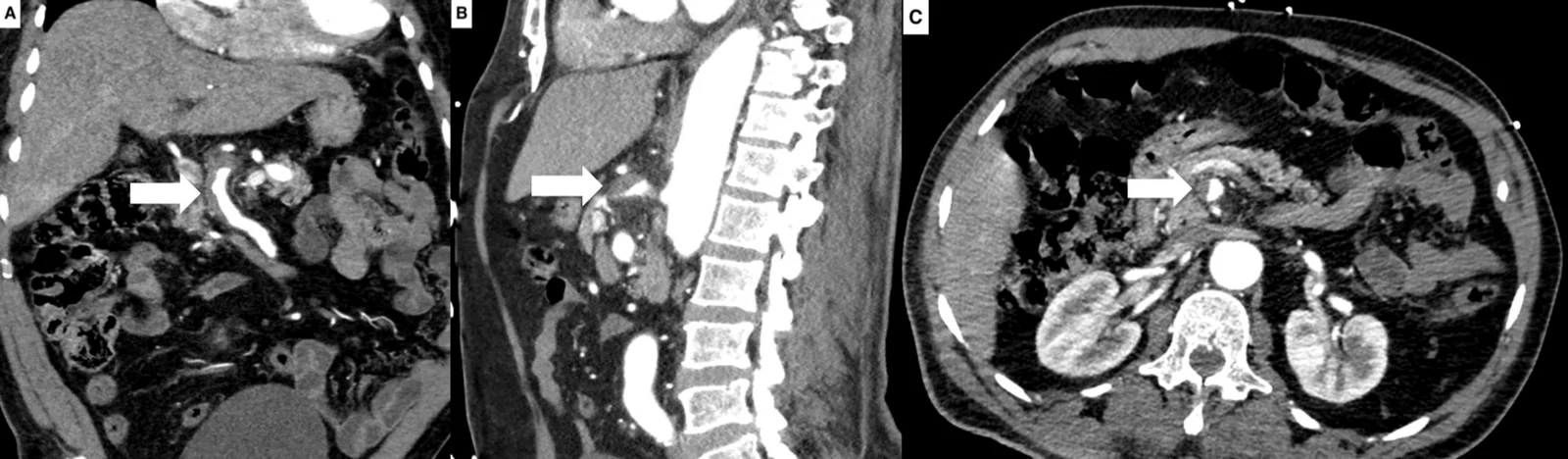
Initial CTA of the abdomen and pelvis on the day of admission demonstrating a superior mesenteric artery dissection measuring approximately 4 cm in length. (A) Coronal, (B) sagittal, and (C) axial views showing the dissection with associated mild-to-moderate luminal narrowing CTA: computed tomography angiography

The initial differential diagnosis included acute mesenteric ischemia, pancreatitis, perforated viscus, aortic pathology, and other causes of an acute abdomen. Prompted by the combination of severe abdominal pain, severe blood pressure elevation, and concern for vascular pathology, computed tomography angiography (CTA) was performed on the day of admission. This revealed an LGA dissection and a 7-mm aneurysm arising from the proximal celiac axis (Figure 2). Furthermore, the SMA was noted to have a 4-cm-long dissection proximally associated with mild-to-moderate narrowing (Figure 3). No evidence of bowel ischemia, perforation, or active hemorrhage was identified. The patient was admitted for close hemodynamic monitoring, and vascular surgery was consulted. Given the absence of ischemia or peritonitis, conservative management was chosen as the mainstay of treatment. Antithrombotic therapy was initiated with aspirin 81 mg and a continuous intravenous (IV) heparin infusion, with a plan to transition to oral anticoagulation in the outpatient setting. Blood pressure control targeted an SBP below 130 mmHg, and hydralazine was administered as needed. He was treated with IV opioids for pain control. Serial abdominal examinations and lactate measurements were performed to monitor for evolving ischemia. The patient was maintained on bowel rest with gentle IV fluid administration.

**Figure 4:**
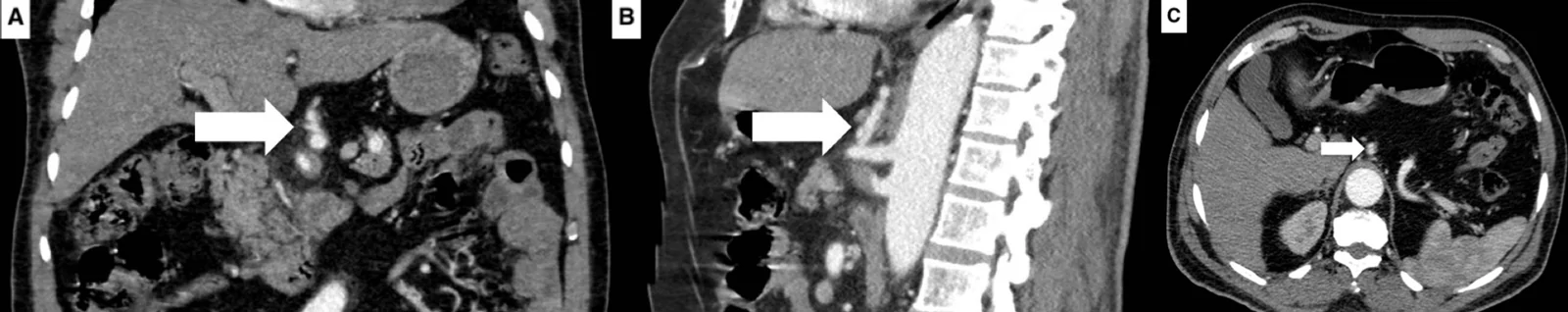
Follow-up CTA of the abdomen and pelvis obtained two days after the initial study, again demonstrating unchanged left gastric artery dissection. (A) Coronal, (B) sagittal, and (C) axial views showing persistent intimal flap and luminal irregularity CTA: computed tomography angiography

**Figure 5:**
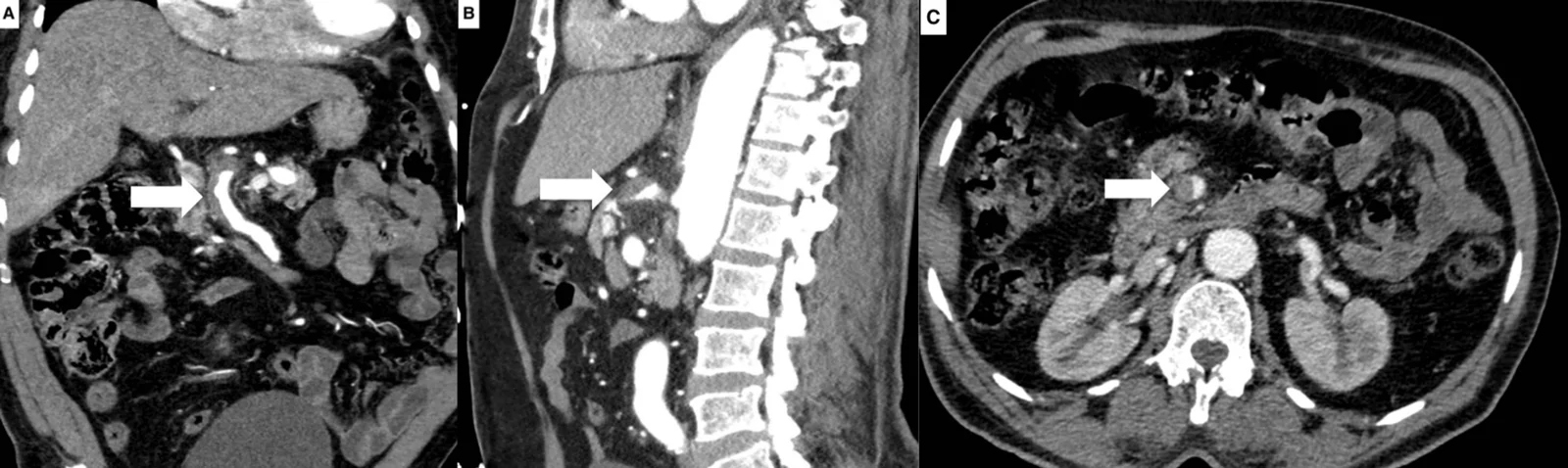
Follow-up CTA of the abdomen and pelvis obtained two days after the initial study, demonstrating a stabilized superior mesenteric artery dissection measuring approximately 4 cm in length. (A) Coronal, (B) sagittal, and (C) axial views are shown CTA: computed tomography angiography

Repeat CTA 48 hours later demonstrated a minimally narrowed SMA lumen and an unchanged LGA dissection with aneurysm (Figures 4, 5). The patient remained hemodynamically stable without clinical or laboratory evidence of bowel ischemia, and his abdominal pain improved with conservative management. He was discharged on hospital day 5 with aspirin 81 mg daily and apixaban 5 mg twice daily for three months, after which aspirin 325 mg daily was planned as a long-term antiplatelet therapy to reduce thromboembolic risk. He was instructed to maintain strict BP control and was scheduled for outpatient vascular surgery follow-up in one month.

## Discussion

SIVAD is a rare clinical entity that most frequently involves the SMA [2]. Involvement of the LGA is exceptionally uncommon, with only a limited number of cases documented in the literature. The concurrent presentation of a chronic LGA dissection and an acute SMA dissection in the same patient is a highly unusual finding; consequently, the natural history of such dual-vessel involvement remains incompletely understood. Hypertension is the most consistently reported risk factor, as chronic shear stress and intimal injury predispose the arterial wall to disruption [4]. Other associated conditions include atherosclerosis, connective tissue disorders, fibromuscular dysplasia, and segmental arterial mediolysis [5].

The radiographic distinction between the acute and chronic dissections in this patient provided crucial insight into the disease chronicity and directly informed our management. The LGA dissection exhibited aneurysmal remodeling, a hallmark of chronic wall degeneration. In contrast, the SMA dissection presented with acute luminal narrowing without evidence of collateralization, correlating with the patient’s sudden onset of abdominal pain. An additional notable feature of this presentation was the transient narrow-complex SVT observed on the initial electrocardiogram. While tachyarrhythmias can theoretically induce abrupt fluctuations in blood pressure and increase arterial shear forces, thereby potentially exacerbating arterial compromise and potentiating dissection, it is equally plausible that the SVT was a secondary physiological response. Further investigation is required to clarify whether a history of SVT directly predisposes individuals to visceral arterial dissection or if it primarily manifests as a reactive phenomenon in these acute settings.

The optimal medical management for uncomplicated SIVAD remains a subject of ongoing debate. While strict blood pressure control is universally recommended to halt dissection propagation, consensus regarding the addition of antithrombotic therapy is lacking. Some authors advocate hemodynamic control alone in asymptomatic patients, whereas others recommend antiplatelet or anticoagulant therapy to prevent false-lumen thrombosis and distal thromboembolization [7,9]. In this case, given concurrent dissections in the absence of ischemia, an antithrombotic strategy was initiated in addition to optimizing blood pressure. This regimen aimed to maintain true lumen patency and mitigate embolic risk during the critical phase of acute vascular remodeling.

Endovascular or surgical intervention in SIVAD is an additional mode of management that may be considered for certain presentations. Patients demonstrating persistent or worsening abdominal pain, evidence of bowel ischemia, rapid aneurysmal enlargement, or deteriorating status despite medical therapy may qualify for stent placement or surgical reconstruction to restore arterial flow and prevent catastrophic complications [9]. In our patient, however, an initial strategy of medical optimization with close outpatient vascular surveillance was justified. This conservative approach was supported by the following important clinical factors: documentation of LGA aneurysmal stability on prior imaging, radiographic evidence of SMA dissection during hospitalization, resolution of abdominal pain without development of peritoneal signs, and optimization of blood pressure. By demonstrating clinical improvement without operative intervention, this case highlights that even rare, multivessel visceral dissections can be successfully managed medically, provided end-organ perfusion remains intact and the patient is closely monitored.

## Conclusions

Concurrent spontaneous LGA and SMA dissections represent an exceedingly rare and diagnostically complex presentation. This case illustrates that acute, severe abdominal pain out of proportion to examination findings in the setting of a hypertensive crisis may warrant prompt cross-sectional vascular imaging to rule out visceral artery dissection and potential bowel ischemia. Even in the presence of high-risk anatomical features such as aneurysmal changes, nonoperative conservative management with aggressive blood pressure control, short-term antithrombotic therapy, and close serial monitoring may achieve favorable clinical outcomes without the need for invasive endovascular or surgical intervention for specific patients. Longitudinal follow-up with surveillance imaging remains essential to monitor vessel remodeling and mitigate the long-term risk of aneurysmal progression or thromboembolic complications.
